# When Predictions Take Control: The Effect of Task Predictions on Task Switching Performance

**DOI:** 10.3389/fpsyg.2012.00282

**Published:** 2012-08-08

**Authors:** Wout Duthoo, Wouter De Baene, Peter Wühr, Wim Notebaert

**Affiliations:** ^1^Department of Experimental Psychology, Ghent UniversityGhent, Belgium; ^2^Institut für Psychologie, Technische Universität DortmundDortmund, Germany

**Keywords:** task switching, proactive cognitive control, expectancy bias, switch cost, prediction

## Abstract

In this paper, we aimed to investigate the role of self-generated predictions in the flexible control of behavior. Therefore, we ran a task switching experiment in which participants were asked to try to predict the upcoming task in three conditions varying in switch rate (30, 50, and 70%). Irrespective of their predictions, the color of the target indicated which task participants had to perform. In line with previous studies (Mayr, 2006; Monsell and Mizon, 2006), the switch cost was attenuated as the switch rate increased. Importantly, a clear task repetition bias was found in all conditions, yet the task repetition prediction rate dropped from 78 over 66 to 49% with increasing switch probability in the three conditions. Irrespective of condition, the switch cost was strongly reduced in expectation of a task alternation compared to the cost of an unexpected task alternation following repetition predictions. Hence, our data suggest that the reduction in the switch cost with increasing switch probability is caused by a diminished expectancy for the task to repeat. Taken together, this paper highlights the importance of predictions in the flexible control of behavior, and suggests a crucial role for task repetition expectancy in the context-sensitive adjusting of task switching performance.

## Introduction

A hallmark of human cognition lies in the ability to proactively anticipate relevant future events and steer both action and perception accordingly. Current influential theories of cognition advance this proactive prediction generation ability as a central mechanism of brain functioning, marking a shift away from the view of the brain passively reacting to incoming stimulation. Predictive representations of both visual (e.g., Bar, 2007; Summerfield and Egner, 2009), auditory (Kumar et al., 2011), and olfactory (Zelano et al., 2011) information have been shown to guide and prepare the brain for a forthcoming stimulus, aiding information processing in a noisy and unpredictable environment. By continuously generating predictions about the environment, the cognitive system is also able to learn and associate specific actions or stimuli with specific outcomes. Learning on the basis of these prediction-driven outcomes is ascribed a central role in optimizing action selection and response execution in recent modeling work (Alexander and Brown, 2011; Silvetti et al., 2011). In line with the conception of the predictive brain, this paper aimed to investigate how self-generated predictions can flexibly steer attentional control through advance preparation, by referring to recent empirical work in the Stroop conflict task (Duthoo et al., submitted) and providing new evidence from a task switching experiment.

Attentional control is typically studied by means of a conflict paradigm, such as the Stroop conflict task (see MacLeod, 1991, for a review). In this task, participants are asked to respond to the color of a color word while ignoring its meaning. As the color and word dimension of the stimulus can either overlap or not, easy (congruent) and difficult (incongruent) stimulus conditions are created, respectively. Optimal task performance requires adaptively adjusting attention to the relevant (color) and irrelevant (word meaning) dimension. In general, these attentional adjustments can be grouped into two categories based on the underlying mechanism and the moment in time they are implemented by the cognitive system (Egner, 2007; Wühr and Kunde, 2008). According to a *reactive* control account, adjustments to the control settings occur in response to the target, corresponding to the metaphor of the reactive brain. Current models typically assume that it is the conflict on a given trial that triggers subsequent control up-regulation, characterized by a strengthening of task-relevant associations (Botvinick et al., 2001; Verguts and Notebaert, 2008, 2009). This theoretical framework has been successfully applied to many attentional control phenomena, including the reduction of the congruency effect following high-conflict incongruent trials in single-task paradigms (i.e., the Gratton effect; Gratton et al., 1992; for a review, see Egner, 2007), but also the increase of the switch cost following high-conflict incongruent stimuli in dual-task paradigms (Goschke, 2000; Braem et al., submitted). Alternatively, control adjustments can also be triggered in anticipation of the upcoming task or target, biasing the task or attentional set proactively. These *proactive* control adjustments, captured by the metaphor of the predictive brain described above, have received considerably less attention in the cognitive control literature.

In order to investigate this type of expectancy-induced control, two different strategies have been pursued. On the one hand, subjects’ expectancies can be manipulated implicitly. Studies on attentional control have, for example, manipulated the proportion of incongruent trials (Logan and Zbrodoff, 1979) or congruency level transitions (Duthoo and Notebaert, 2012) to induce preparatory strategic control adjustments. Whereas the first manipulation successfully triggered anticipatory control, reflected in faster reactions to highly expected incongruent trials than to unexpected congruent trials, the second, more subtle manipulation appeared not strong enough to elicit expectancy-induced adaptation effects that were clearly dissociable from reactive, conflict-induced adjustments (see also Jiménez and Méndez, 2012). Alternatively, a more common and widespread experimental tool to probe anticipatory control adjustments is to cue participants explicitly about the upcoming stimulus event (for some early experimental work with the cueing paradigm, see Neill, 1978; Logan and Zbrodoff, 1982; Harvey, 1984). More recently, Aarts and Roelofs (2011) applied a probabilistic cueing procedure to a Stroop-like task to point out that the anticipation of upcoming conflict (or lack of conflict) can trigger similar sequential adjustments as experienced conflict (or lack thereof) on the previous trial, both behaviorally and in the activation pattern of the anterior cingulate cortex (ACC). In similar vein, Correa et al. (2009) found that anticipating conflict in a cued congruency task sped up both conflict detection and conflict resolution.

However, investigating proactive control by means of a cueing paradigm is not really testing the implications of a predictive brain, as it is assumed that we constantly generate predictions ourselves. Compared to the large amount of studies concerning cue-induced attentional control, few studies have centered on the effect of self-generated predictions on subsequent processing. Yet, human predictive behavior itself has been the focus of much experimental work outside the field of cognitive control. Interestingly, an influential line of research revealed that people’s predictions and expectancies are often strongly biased (e.g., Kahneman et al., 1982), as they either overestimate or underestimate the actual probability of events to occur (see also Ayton and Fischer, 2004). When confronted with a random run of stimuli, participants will typically indicate that longer runs of a particular event have to be balanced out by the occurrence of the alternative event, a phenomenon known as the gambler’s fallacy. This tendency for negative recency is also typically found when people are asked to generate or identify a random sequence (see Nickerson, 2002, for a review). However, other studies have shown that people can also display the opposite expectancy bias, the tendency to predict positive recency. A study of Kareev (1995), for example, in which participants were asked to predict the next item on the list, revealed that subjects typically overestimate repeating events. According to Kareev, this repetition bias stems from a persistent tendency to perceive or find patterns and causality in the environment (note, however, that the same tendency, seen from another perspective, can also result in probability matching behavior at the outcome level, the strategy to predict the events in proportion to their probability of occurrence; see Gaissmaier and Schooler, 2008). Apart from its impact on simple serial two-choice reaction time tasks (Remington, 1969; Soetens et al., 1985), the impact of this expectancy bias on information processing and attentional control remains still relatively uninvestigated. Given both these persistent prediction biases and the cognitive system’s inherent prepotency to generate predictions and evaluate its outcomes, investigating self-generated expectancies and comparing their impact on subsequent processing to that of exogenously triggered expectancies might reveal new insights into how the brain implements proactive control.

In a previous study (Duthoo et al., submitted), we undertook a first attempt to measure these biased predictions explicitly and verify their influence on cognitive control by subjecting participants to a Stroop task and letting them predict the congruency level of the upcoming Stroop stimulus. Interestingly, after recoding participants’ predictions (“Do you expect a congruent or incongruent trial?”) relative to the congruency level of the previous trial, results revealed a clear repetition bias in the prediction pattern: in line with Kareev (1995), participants expected the congruency level to repeat from one trial to the next in 65% of all cases, even though congruency level repetition probability was set at 50%. Moreover, attentional adjustments (i.e., a Gratton effect) were only found when they anticipated a congruency level repetition. Participants showed both a reduced interference of repeating conflict trials (by proactively narrowing attention to the stimulus color) and increased facilitation of repeating non-conflicting trials (by proactively allowing the word meaning to influence response selection). In case of an unexpected congruency level alternation, these preparatory adjustments backfired and longer reaction times were registered, resembling the results of Aarts and Roelofs (2011) in a probabilistic cueing experiment. Interestingly, analyses of the congruency alternation predictions also suggested that in anticipation of an alternation, participants seemed to switch to a default control mode, as no sequential adjustments were found. In sum, the study revealed a clear bias toward predicting repeating events, and an optimization of control processes (i.e., a Gratton effect) in anticipation of such repeating events. Alternation expectancies, on the other hand, did not induce preparatory control.

Contrary to the literature on conflict control, the contribution of a preparatory component in task switching research has played a central role in the theoretical debate (e.g., see Karayanidis et al., 2010 and Kiesel et al., 2010 for recent overviews), overshadowing research on the reactive priming effects of the previous task-set on current task performance. In order to investigate these proactive adjustments, similar strategies have been implemented, aimed at inducing expectancies either implicitly or explicitly. As an example of the former strategy, fixed (predictable) task sequences (i.e., the alternating-runs paradigm; Rogers and Monsell, 1995) have been introduced to compare predictable task switch trials to predictable repetition trials. Even though two simple tasks were used and the task sequence was entirely predictable, this paradigm consistently evoked increased reaction times and higher error rates on switch compared to repetition trials (i.e., robust switch costs). To probe the impact of explicit expectancies on these switch costs, the explicit cueing paradigm (Meiran, 1996) was developed, in which cues specified the required task in a random run of task repetitions and switches. This cueing paradigm has been extensively used to evidence preparatory reductions in switch costs (e.g., Meiran, 1996; Koch, 2003), albeit not without its own set of methodological pitfalls (see Logan and Bundesen, 2003; but see also De Baene and Brass, 2011 and Jost et al., submitted).

Whereas a previous single-task study (Duthoo et al., submitted) suggested that alternation expectancies did not induce preparatory control adjustments, research on task switching has convincingly shown how increasing the preparation interval prior to an anticipated task alternation led to more controlled processing (i.e., a reduced switch cost). Monsell et al. (2003), for example, reported performance benefits for predictable compared to unpredictable task switches, suggesting that participants can strategically control their task-set readiness in function of their expectation, and, more precisely, in function of the probability of encountering a task switch on the upcoming trial. In similar vein, further research has robustly found a reduced switch cost with increasing switch probability (Mayr, 2006; Monsell and Mizon, 2006; Schneider and Logan, 2006; Bonnin et al., 2011). Others have pointed out that not only when expecting a task alternation, but also in anticipation of an expected task repetition, task-set readiness can be adjusted for optimal task performance, resulting in strong repetition benefits (Dreisbach et al., 2002). In sum, more so than in single-task paradigms, dual-task performance seems to rely on a strong anticipatory control component.

Even though the theoretical debate about this anticipatory control component is still ongoing, a key role is usually attributed to repetition expectancy. For example, the smaller difference between switch and repeat trials in a context with a 50% compared to a 30% switch probability is sometimes explained by the fact that participants match their task preparation to the probability of the switch and repeat conditions, thus equally preparing both tasks in a 50% switch probability context (Dreisbach et al., 2002; Brass and von Cramon, 2004; Monsell and Mizon, 2006). Alternatively, other authors suggested that people prepare the other task on part of the trials (e.g., Monsell and Mizon, 2006), resulting in extra preparation and thus longer reaction times on task repetition trials (when their guess was wrong) and less preparation and thus faster reactions to task switch trials (when their guess was right). Importantly, both explanations stress the importance of expectancies about the upcoming task. However, as indicated above, past research has consistently found that people’s predictions are biased and therefore often do not match the actual probability in a given context (especially in the context of a random sequence of events; but see the work of Gaissmaier and Schooler, 2008, showing that the search for patterns can also result in probability matching at the outcome level). Moreover, the abovementioned studies never measured expectancies themselves, so that it remains a question for further research how expectancies can steer task preparation.

To shed some light on this issue, as well as to compare self-generated predictions in a dual-task paradigm to previous findings in a single-task paradigm, we decided to apply a similar procedure as our previous study on prediction-driven adjustments in the Stroop task (Duthoo et al., submitted). Therefore, we asked participants to try to predict the upcoming task on a trial-by-trial basis in one of three between-subjects conditions varying in switch rate (30, 50, and 70%), and probed both how these contexts affected the prediction pattern and how these predictions themselves influenced the task switch cost. Similar to our previous findings in the Stroop task, we expected predictions to evoke advance preparation for the upcoming target. More specifically, we expected repetition predictions to induce a strong reaction time benefit when a task repetition was actually presented, and a huge cost when one had to unexpectedly switch tasks, irrespective of condition. In contrast with the strong switch costs (and repetition benefits) following repetition predictions, we expected that alternation predictions evoke less strong preparatory effects (Duthoo et al., submitted), thereby reducing the switch cost, irrespective of condition. Consequently, assuming that participants’ tendency to predict task repetitions is attenuated with increasing switch probability, we predicted to replicate the finding of a reduced switch cost in contexts of higher switch probabilities (Mayr, 2006; Monsell and Mizon, 2006; Schneider and Logan, 2006).

## Materials and Methods

### Participants

Forty-eight Ghent University students (14 males; age: 17–28) signed up to participate in one of the three conditions (*n* = 16) of the experiment, lasting approximately 45 min. They received a monetary payment in return. Prior to the testing, participants provided written informed consent.

### Stimuli and apparatus

A program written with T-scope software (Stevens et al., 2006) controlled the experiment. All stimuli were displayed on a 17-inch monitor, with a viewing distance of approximately 50 cm. The numbers 1–9, with the exclusion of 5, served as the target stimuli, presented in Arial, font size 32. These stimuli were presented centrally on a black background in yellow (for the magnitude task) or blue (for the parity task). Responses were registered by means of a QWERTY keyboard.

### Design and procedure

Participants were randomly assigned to one of the three experimental conditions, differing only in the amount of task switches during the three blocks where an explicit task prediction was registered. In the *repetition condition*, the task switch probability was restricted to 30%. In the *intermediate condition*, participants were confronted with an equal amount of task repetitions and alternations (50%). The *alternation condition* increased the task switch probability to 70%.

Throughout all blocks of the experiment, each target number was equally often presented in blue and yellow, implying that within each block participants performed an equal amount of magnitude and parity judgments. Selection of the target number was pseudo-random, with the restriction that each of the eight possible number targets appeared an equal amount of times in each of the two possible colors within one block. In all dual-task blocks, consisting of 80 trials, each target number was thus presented five times in both blue and yellow. Participants had to respond by pressing the E or U keyboard key for small or even target numbers and the R or I keyboard key for large or odd target numbers. The mapping of the task (magnitude or parity) to the middle and index finger of the left hand (keys E and R, respectively) or index and middle finger of the right hand (keys U and I, respectively) was counterbalanced across participants. In order to indicate which of the two tasks they expected, participants had to press the V or N key with their thumbs. The mapping of these keys to either a magnitude or parity task prediction was compatible with the mapping of the left or right hand to one of the two tasks.

In all conditions, participants were first trained on each of the two tasks separately during 40 trials of first magnitude and then parity judgments, adding up to 80 single-task practice trials. Hereafter, the two tasks where combined during two blocks of 80 trials, as to familiarize participants with the dual-task procedure. For these dual-task training blocks the task switch probability was kept at 50% in all three conditions. The color in which targets were presented indicated the task participants had to perform. A yellow number target asked for a magnitude judgment, whereas a blue target required a parity response. In the final phase of the experiment, three blocks of 80 trials were presented during which participants first had to predict which of the two tasks they expected to come next. Irrespective of their choices, the color in which the upcoming target was presented again indicated which of the two task participants had to perform, thereby serving as a feedback signal for their task predictions. For their performance on the target numbers no error feedback was provided. A store coupon was promised to the participant who performed best in the three last blocks for each condition, taking into account both the amount of correct predictions and mean reaction times and error percentages. In between blocks, participants took a short, self-paced break. After completing the experiment, participants filled in a short questionnaire, probing their awareness of the switch probability manipulation and their use of strategies in predicting the task sequence.

Each trial started with the presentation of a fixation cross for 500 ms. In the training blocks, this was followed by the target, which appeared on the screen until a response was registered, with the maximal reaction time restricted to 2500 ms. Next, the screen turned black for 500 ms, serving as the inter-trial-interval. In trials in which participants also had to predict the task on the next trial, a fixation cross was first presented for 500 ms, after which an instruction appeared on the screen (“Next trial?”) that remained visible on the screen until participants clicked one of the two designated keyboard keys. Hereafter, a fixation cross was again displayed for 500 ms, after which a number target appeared on the screen, with identical timing values as described above.

## Results

In the results section, we focus on the three experimental blocks in which predictions were also registered. Two participants who did not engage in the prediction task (by “predicting” the same task throughout at least one of the three experimental blocks) were removed from the analysis, restricting the number of participants in the intermediate and alternation condition to 15. Non-responses and badly recorded data (adding up to 1.6%) were excluded from both the reaction time and performance error analysis. We applied the multiple comparison correction method put forward by Holm (1979) in order to control for the family wise error rate, adjusting the *p*-values of the post tests in the reaction time and error analysis accordingly.

### Reaction times and predictions

Before conducting the reaction time analysis, the data were subjected to a trimming procedure. We first excluded the trials on which participants committed an error (8.1% of the remaining data; distributed equally over the three conditions). Hereafter, the first trial of each block and RT outliers (±2.5 SD, calculated separately per condition, subject, and task) were removed (another 3.9%). Taken together, the analysis was thus carried out on 86.9% of the complete data.

**Figure 1:**
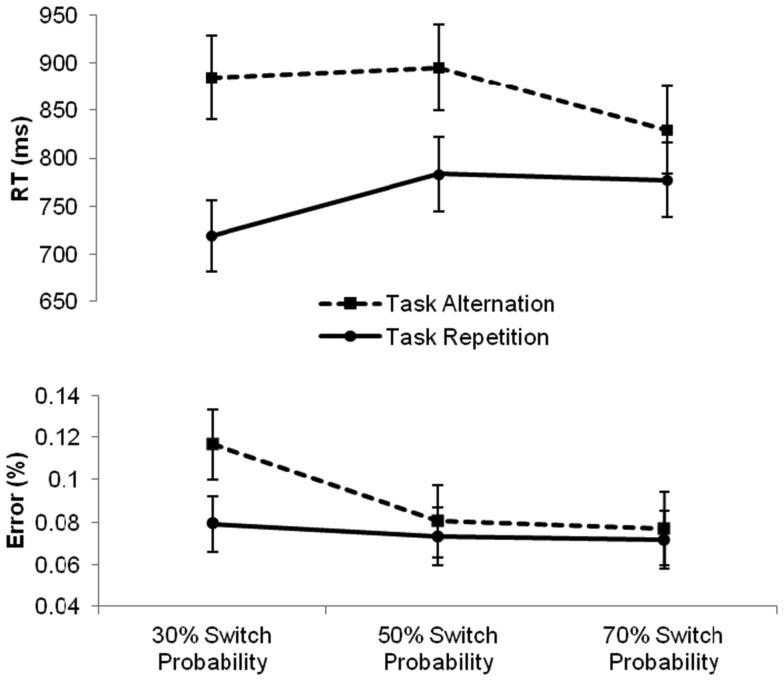
**Mean reaction times (RTs, in milliseconds) and error percentages for task repetitions (full line) and task alternations (dashed line) in the three conditions varying in switch probability**. Error bars represent 95% confidence intervals around the mean.

First, a mixed-design analysis of variance with the between-subjects variable Condition (three levels: repetition, intermediate, and alternation) and the within-subjects variables Task (two levels: magnitude and parity) and Sequence (two levels: repetition and alternation) was carried out. Results revealed main effects of Task, *F*(1, 43) = 57.36, *p* < 0.0001, reflecting faster magnitude than parity judgments (757 and 877 ms, respectively) and Sequence, *F*(1, 43) = 116.95, *p* < 0.0001, indicating the presence of a switch cost of 106 ms, but not a main effect of Condition, *F*(2, 43) < 1, ns. The two-way interaction between Task and Sequence turned out significant as well, *F*(1, 43) = 5.47, *p* < 0.05, reflecting a larger switch cost for the parity task compared to the magnitude task (120 and 93 ms, respectively), irrespective of Condition, *F*(2, 43) < 1, ns. Most importantly, the analysis revealed a two-way interaction between Sequence and Condition, *F*(2, 43) = 11.05, *p* < 0.0001, implying that the size of the switch cost was significantly affected by the transitional manipulation. Further independent-samples *t*-tests showed that, compared to the switch cost of 112 ms in the intermediate condition, the switch cost was significantly reduced to 52 ms by increasing the switch probability in the alternation condition, *t*(28) = 3.5, *p* < 0.01. Decreasing the switch probability to 30% in the repetition condition significantly increased the switch cost to 166 ms compared to the alternation condition, *t*(29) = 4.5, *p* < 0.0001. The increase in switch cost of 54 ms in the repetition compared to the intermediate condition was only marginally significant, *t*(29) = 2.0, *p* = 0.056. These differences in the switch cost over conditions are depicted in Figure 1.

Next, we took a deeper look into participants’ task prediction patterns. Irrespective of condition, participants predicted the magnitude and parity task equally often (i.e., 50%, on average, SD = 5.4%). These task predictions were then recoded into repetition or alternation predictions, relative to the task presented on the previous trial. In line with our manipulation of task switch probability, participants in the repetition condition predicted more task repetitions (78%), both compared to participants in the intermediate [66%, independent-samples *t*(29) = 3.1, *p* < 0.001] and participants in the alternation condition [51%, independent-samples *t*(29) = 7.63, *p* < 0.0001]. Remarkably, in all three conditions a task repetition bias was found, as comparisons between the task switch prediction rate and the actual task switch probability indicated that both in the intermediate condition [66% compared to 50%, *t*(14) = 6.77, *p* < 0.0001], repetition condition [78% compared to 70%, *t*(15) = 2.86, *p* < 0.05] and alternation condition [51% compared to 30%, *t*(14) = 8.39, *p* < 0.0001] the amount of task repetitions was consistently overpredicted.

**Figure 2:**
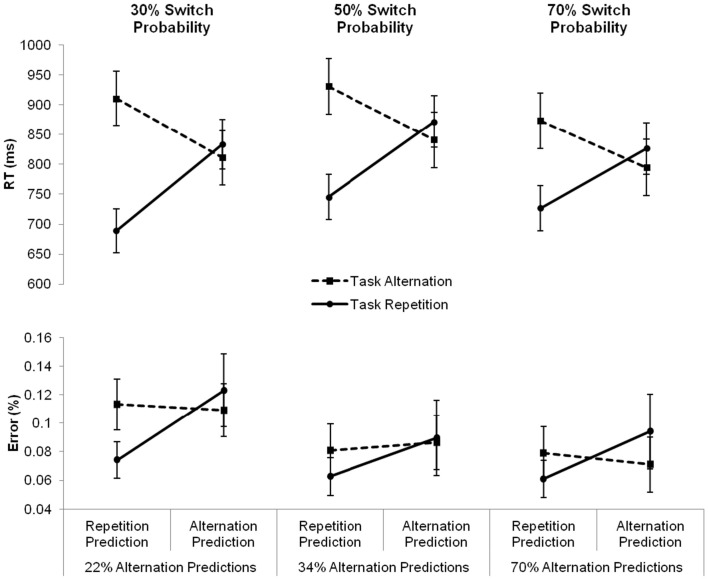
**Mean reaction times (RTs, in milliseconds) and error percentages for task repetitions (full line) and task alternations (dashed line) following repetition and alternation predictions, separately for the three conditions varying in switch probability**. Under each of the graphs, the corresponding overall percentage of alternation predictions is presented. Error bars represent 95% confidence intervals around the mean.

Finally, we examined the effect of these task predictions on task performance, by investigating how repetition and alternation expectations impacted the switch cost. To this end, we ran a mixed-design analysis of variance with the between-subject variable Condition (three levels: repetition, intermediate, and alternation) and the within-subjects variables Prediction and Sequence (two levels: repetition and alternation)^1^. Apart from the main effect of Sequence, *F*(1, 43) = 59.89, *p* < 0.0001, reflecting a switch cost, the analysis also revealed a marginally significant main effect of Prediction, *F*(1, 43) = 3.87, *p* = 0.056, indicating that number targets were responded to 17 ms slower following alternation predictions than following repetition predictions. Importantly, a significant interaction between Prediction and Sequence was also found, *F*(1, 43) = 88.75, *p* < 0.0001. The three-way interaction with Condition did not reach significance, *F*(2, 43) < 1, ns, suggesting that participants’ predictions influenced the switch cost similarly in all three conditions. Following an alternation prediction, the switch cost, calculated as the difference between an expected task alternation and an unexpected task repetition, disappeared completely. Even though inspection of the reaction times suggested a switch benefit numerically (24, 31, and 32 ms in the repetition, intermediate, and alternation condition, respectively), post tests indicated that this difference did not reach statistical significance in any of the conditions (all *p*s > 0.62). Following a repetition prediction, a huge and significant repetition benefit, calculated as the difference between an unexpected task alternation and an expected task repetition, was found in all conditions (222, 116, and 147 ms in the repetition, intermediate, and alternation condition, respectively; all *p*s < 0.0001). This pattern of reaction times is visualized in Figure 2.

### Error rates

First, we ran a repeated-measures ANOVA with the between-subjects variable Condition (three levels: repetition, intermediate, and alternation) and the within-subjects variables Task (two levels: magnitude and parity) and Sequence (two levels: repetition and alternation) on the aggregated error scores. Similarly to the reaction time analysis, we found main effects of Task, *F*(1, 43) = 36.61, *p* < 0.0001, reflecting worse performance on parity than magnitude judgments (12 and 4.6%, respectively) and Sequence, *F*(1, 43) = 9.51, *p* < 0.01, indicating higher error rates on task alternations than on task repetitions (9.2 and 7.4%, respectively), but no main effect of Condition, *F*(2, 43) < 1, ns. The two-way interaction between Task and Sequence also reached significance, *F*(1, 43) = 5.07, *p* < 0.05, indicating that switching to a parity task (compared to repeating this task) increased the error rate (3.2%), whereas switching to a magnitude task did not. Most importantly, we again found a significant interaction between Sequence and Condition, *F*(2, 43) = 3.22, *p* < 0.05, indicating that the size of the error switch cost differed significantly between the three conditions, irrespective of task, *F*(2, 43) < 1, ns. Further independent-samples *t*-tests revealed that this interaction was brought about by a significant increase in the error switch cost (3.7%) in the repetition condition compared to the intermediate condition, *t*(29) = 2.09, *p* < 0.05, whereas the error switch cost was not statistically lower in the alternation condition compared to the intermediate condition, *t*(28) < 1, ns. The error rates for task repetitions and task alternations in each of the three conditions are visualized in Figure 1.

In order to investigate how participants’ predictions had an impact on the error rates, we conducted another repeated-measures ANOVA with the between-subjects variable Condition (three levels: intermediate, repetition, and alternation) and the within-subjects variables Prediction and Sequence (two levels: repetition and alternation). This analysis revealed only a main effect of Prediction, *F*(1, 43) = 5.73, *p* < 0.05, indicating that an alternation prediction produced more erroneous responses compared to a repetition prediction (9.6 and 7.8%, respectively). The two-way interaction between Prediction and Sequence was only marginally significant, *F*(1, 43) = 3.3, *p* = 0.076. The data pattern closely resembled the reaction pattern, showing a trend for the error switch cost to be absent following alternation predictions, and present following repetition predictions. Again, this pattern did not differ significantly between the three conditions, *F*(2, 43) < 1, ns. The error rates for task repetitions and task alternations following repetition and alternation predictions in each of the three conditions are presented in Figure 2.

## Discussion

In the present study, we aimed to investigate how self-generated predictions influence conflict and task control, expanding previous research on expectancy-induced proactive control. To do so, we inserted explicit task predictions into a task switching procedure, thereby complementing as well as elaborating on a previous experiment in which the influence of congruency level predictions on subsequent Stroop performance was put to the test (Duthoo et al., submitted). Results revealed three interesting findings.

Firstly, analysis of participants’ prediction patterns exposed a bias toward predicting task repetitions in all three conditions. In the intermediate condition, in which the two tasks alternated in 50% of all transitions, participants displayed a clear task repetition bias (66%). Also in the alternation condition, participants still predicted a task repetition in 51% of all transitions, when only 30% were actually presented. Moreover, reaction times and error rates showed that irrespective of condition, reactions following a task alternation prediction were slower and more error-prone. At first sight, this tendency to predict repeating stimulus events, or “hot hand fallacy,” might seem at odds with the literature on probability matching (Gaissmaier and Schooler, 2008), revealing participants’ tendency to match their choice behavior to the actual probability of two stimuli that are not equally likely to be presented. Yet, given that participants in the current experiment were asked to predict the upcoming task rather than the task transition, participants matched probabilities quite well, as irrespective of condition the two tasks were predicted equally often (i.e., 50%). Still, further insight into the transitional probabilities could help them predicting the upcoming task more accurately. Yet, these transitional probabilities were less readily picked up, since the experiment revealed a clear bias toward expecting repetitions. Interestingly, participants’ prediction error rate only dropped from 50 to 38% in the repetition condition [*t*(15) = 8.9, *p* < 0.0001], in which transitional probability was in line with their repetition expectancy bias.

Secondly, our manipulation of switch probability affected the switch cost as predicted: compared to the switch cost in the intermediate condition with a 50% switch probability, increasing this switch probability decreased the switch cost significantly, whereas decreasing the switch probability strongly amplified the switch cost. Put differently, the switch cost is attenuated under conditions of high switch probability, replicating previous studies (Mayr, 2006; Monsell and Mizon, 2006; Schneider and Logan, 2006; Bonnin et al., 2011). Moreover, results also revealed that switching to the parity task came at a greater cost than switching to the magnitude task, both in reaction time and accuracy. This corresponds well with previous research on asymmetries in switch costs showing that separating the response set of the two tasks results in greater costs in switching to the more difficult task (Yeung and Monsell, 2003). In the current experiment, response set overlap was reduced in terms of response decisions (parity versus magnitude judgments) and stimulus-response mapping (both tasks were mapped to separate hands). Most importantly, this task asymmetry did not interact with predictions, which formed the main focus of this study.

Thirdly, by inserting explicit predictions into the dual-task procedure, we were able to identify a potential mechanism underlying the finding of reduced switch costs in conditions with high switch probability. In all three conditions, the same prediction-driven behavioral adjustments were found: following an alternation prediction, the difference between repetition and switch trials disappeared, whereas repetition predictions were followed by a large switch cost (or a large repetition benefit). Participants in the alternation condition expected more alternations, thereby reducing the switch cost significantly. In other words, the reduction in switch cost in a context of high switch probability might stem from proactively switching to a more controlled processing strategy when expecting task alternations. However, preparing for a task alternation still comes at a cost, as comparisons between correctly predicted task repetitions and alternations revealed a significant residual task switch cost (all *p*s < 0.001). This finding is in line with studies using the explicit cueing paradigm that consistently show that even validly cued task alternations robustly slowed down responses compared to validly cued task repetitions (Meiran, 1996).

On an important note, part of the speed-up in reaction time following correct predictions might reflect an effect of hand priming, as in the current design correct predictions involved the finger of the same hand needed for subsequent task execution, whereas incorrect predictions entailed a switch of hand (e.g., Cooper and Marí-Beffa, 2008). Still, this definitely cannot account for the whole pattern of findings, since predicting the other task relative to the task on the previous trials correctly (i.e., a task alternation in which the same hand was used for predicting and responding to the target) did not produce reactions that were significantly faster than following incorrect task alternation predictions, in which the task repeated but the hand used for predictions differed from the hand used for responding to the target. Taken together, this study suggests that in a dual-task environment, participants expect the task to repeat, leading to improved performance when it does and a large cost when it alternates. Still, in anticipation of a task alternation, participants respond equally fast to a task alternation as to a task repetition. These conclusions are clearly in line with a proactive, expectancy-based account of task switching.

Moreover, the current findings allow drawing interesting parallels between this experiment and the aforementioned previous Stroop experiment, both in the patterns of self-generated expectancies as in their effect on subsequent processing. Compellingly, we found a robust bias toward overpredicting repeating events that was also present in congruency level predictions in the Stroop task. This bias toward expecting task repetitions coincided with a clear processing benefit for these repetition predictions, as alternation predictions typically induced higher errors rates and increased reaction times, irrespective of condition. Interestingly, the observation of reaction time benefits following repetition expectations but not after alternation expectations also bears a striking resemblance to findings within the voluntary task switching paradigm (Arrington and Logan, 2004). In this paradigm, participants can choose which task to perform on a series of bivalent stimuli, with the instruction to perform both tasks equally often. In line with the inherent bias toward repetitions defended in this paper, Arrington and Logan found that the subjects produced more task repetitions (i.e., 68%) than expected if the tasks were performed in a pure random sequence. Moreover, deliberately choosing to switch tasks slowed down task performance significantly (i.e., a significant *switch cost* was found). Taken together, the experiment revealed that participants displayed a clear reluctance to switch tasks.

Similar to the voluntary task repetition and switch decisions, repetition and alternation predictions clearly produced a differential effect on subsequent processing: repetition predictions were followed by a strong reaction time benefit when an actual task repetition was presented, and a large cost when one then had to (unexpectedly) switch. Again, this pattern closely resembled findings in our previous Stroop study (Duthoo et al., submitted), where a clear congruency level repetition benefit and congruency level alternation cost were found following repetition predictions. Yet, whereas congruency level alternation predictions were not followed by behavioral adaptations in the Stroop task, the current experiment showed that following task alternation predictions the difference between an actually presented task alternation and an unexpected task repetition disappeared.

Crucially, this pattern of results did not differ between the three conditions varying in switch probability. Therefore, the present experiment suggests an explanation for the often replicated finding of reduced switch costs in conditions with a higher switch probability (Mayr, 2006; Monsell and Mizon, 2006; Schneider and Logan, 2006): increasing the switch probability increases the expectancy for task alternations, which was found to be followed by a reduction in the switch cost. However, the interpretation of this reduced switch cost in anticipation of a task alternation is still open to debate.

One possible explanation, as was also put forward by Monsell and Mizon (2006), is that participants adopt a “neutral control state,” right in between the two task-sets. When the color of the target then indicated which of the two task-sets was appropriate, reactions to either one of the two tasks would be equally fast. This is exactly the pattern of results we found following alternation predictions, and it emerged in all three conditions. Moreover, this corresponds well with the absence of sequential modulations of the Stroop effect following congruency level alternation predictions, which was also explained by participants adopting a “neutral control mode” (Duthoo et al., submitted).

Alternatively, one can assume that both repetition and alternation predictions lead to advance preparation of the upcoming task, yet preparation for task alternations is never complete (i.e., there is a residual switch cost, e.g., Meiran et al., 2000). Also in our experiment, correctly predicted task alternations were responded to much slower than correctly predicted task repetitions, irrespective of condition. In case of a correctly predicted task alternation, advance preparation speeds up responding compared to an unexpected task alternation (i.e., following a task repetition prediction). Yet, because of a residual switch cost, these reactions are not significantly faster than those to unexpected task repetitions (i.e., following a task alternation prediction), where preparation misfires, but no residual switch cost affects performance. The same logic holds if one assumes the difference between switch and repeat trials to arise from adaptation to the task-set on repetition trials, reflected in a repetition benefit, rather than from reconfiguration of the task-set on switch trials, reflected in a (residual) switch cost (De Baene et al., 2012). In the case of an unexpected task repetition following a task alternation prediction, reaction times will be relatively slower than for expected task repetitions, yet equally fast to an expected task alternation, where no task-set adaptation benefit was present. However, the current data do not allow differentiating between the adaptation and reconfiguration view, as both predict the same data pattern: following correct repetition predictions, both preparation and task-set adaptation (or lack of reconfiguration) will speed-up an actual task repetition, whereas following correct alternation predictions, preparation, and the lack of task-set adaptation (or need for reconfiguration) have effects in opposite directions, explaining the intermediate reaction times. Whether this explanation in terms of equal preparation for switch and repeat trials following both types of predictions is to be favored over an explanation in terms of a lack of specific preparation for alternation predictions (i.e., a neutral control mode) is an interesting question for future research.

Yet, the current experiment applied a 1:1 mapping between the cue (i.e., the color of the target) and the task (i.e., a magnitude or parity judgment), so that task repetitions were confounded with repetitions of the cue. Therefore, this design does not allow teasing apart the facilitatory effect of repeated-cue-encoding in task repetitions from the effect of executive control processes reconfiguring the cognitive system in task alternations. In order to disentangle cue repetitions from task repetitions, some previous studies have introduced multiple cues per task (e.g., Logan and Bundesen, 2003; Mayr and Kliegl, 2003; see Schneider and Logan, 2011, for a comparison between 1:1 and 2:1 cue-to-task mappings). This approach has led to a rich body of empirical evidence showing that repetition priming of cue encoding is indeed an important component of task switching. Note, however, that these studies have also demonstrated that there are usually also substantial “true” task switch costs remaining (for a review of this evidence, see Jost et al., submitted).

Important in the light of the current results is a study of Schneider and Logan (2006), in which this 2:1 cue-to-task mapping was combined with a transitional probability manipulation similar to ours. In line with the current findings, switch costs were smallest in the condition with a high switch probability and largest when the amount of task repetitions was increased. Modeling of their data led these authors to conclude that the difference in the switch costs between different frequency conditions reflected (automatic or strategic) priming of cue encoding for the frequent transitions. Therefore, an interesting avenue for future research lies in combining a 2:1 mapping strategy with our prediction manipulation to elucidate whether the prediction-driven adjustments in task switching performance reported in this paper were driven by facilitating the speed of cue encoding rather than by promoting advance configuration of task-set.

Given the emphasis recent theories place on prediction-driven adjustments in brain functioning, the paradigm to assess self-generated predictions and probe their impact presented in the current article seems a particularly promising tool for further research. Applying this method, we were able to pinpoint structural biases in human predictions and measure their influence on subsequent processing in a direct way, rather than inferring explanations in terms of expectancy indirectly from the data. Yet, one outstanding question remains whether participants will make similar predictions when they are not explicitly asked to generate them, and, consequently, to what extent these expectancy-driven attentional adjustments can also be found in “normal” Stroop or dual-task behavior.

In conclusion, the research presented in this paper advocated viewing the brain as a predictive rather than a purely reactive device. In this light, the overestimation of repeating events (also referred to as “the hot hand fallacy”) should not necessarily be considered as a weakness of our predictive brain. In real life, there is a much stronger correlation between sequential events than in our artificial lab tasks. For instance, when the road is slippery because of wet conditions in one turn, it is usually a good idea to predict that also the next turn will be slippery and adjust accordingly. It therefore appears adaptive that the cognitive system is more readily optimized in anticipation of a repeating event. This is reflected in a strong repetition benefit for both congruency level and task repetitions. Yet, when interpreting the lack of conflict adaptation and the reduced difference between task repetition and alternations following alternation predictions in terms of participants adopting a neutral control mode, it remains an extremely interesting question to what extent our brain can also prepare for expected changes.

## Conflict of Interest Statement

The authors declare that the research was conducted in the absence of any commercial or financial relationships that could be construed as a potential conflict of interest.

## Footnotes

^1^We did not include the variable Task in this analysis, as this would cause some of the cells of the ANOVA to be calculated on a very limited amount of observations (for instance: a switch to the parity task following an alternation prediction in the repetition condition). We therefore collapsed observations over the two tasks. Still, running the analysis with the Task variable included did not change the pattern of the results. Importantly, the Task variable did not interact significantly with any of the other variables (all *p*s > 0.14).
